# To Dr. Batty

**Published:** 1805-08-01

**Authors:** Thomas Denman


### Medical and Physical Journal.

VOL. XIV.] AUGUST 1, 1805. [NO. LXXVIII.

*Printed for R. PHILLIPS, by W. Thorne, Red Lion Court, Fleet Street, London.*

### To Dr. BATTY.

DEAR SIR,

HAVING been lately informed of two cases of children losing their lives in consequence of cutting what is called the *bridle* of the tongue, by which a fatal hæmorrhage was occasioned, I hope you will excuse my requesting you to insert the following observations on the subject in the Medical and Physical Journal.

It was formerly an almost universally received opinion, that every child was born tongue-tyed. This was a great error; but if a child from mismanagement, or any cause, was incapable of sucking, or indisposed to suck at particular times, or did not suck in the manner the nurse thought right, it was immediately said to be tongue-tyed; and the *frænum* of the tongue being on inspection observed, the opinion was confirmed. All medical men know that the *frænum* of the tongue is a natural part, intended for the important purposes of preventing the retraction of the tongue beyond a certain degree, and for regulating its actions, particularly that of duly modulating its voice. By cutting the frænum, it cannot be doubted but that these advantages are impaired, the enunciation injured, and that which is called speaking thick, pleno ore, is occasioned.

The *frænum* of the tongue is much shorter in some children than in others; but experience has sufficiently proved that when it is apparently short, it will by frequent action be stretched so much as to allow all the necessary and proper motions of the tongue; and even if the *frænum* should confine the end of the tongue, forming a small indentation at the extremity, the child would not be hindered from sucking or speaking distinctly, or suffer any inconvenience from it.

An infant can only use its tongue for sucking, for swallowing its food, and for crying; and the last of these proves that it is not tongue-tyed. Nor have I ever met with one instance of a child, in whom it was absolutely necessary to cut the *frænum* of the tongue. I have indeed sometimes done it to satisfy the prejudice of parents, or those who have the care of children; or to prevent its being done by those who might be less careful than myself; but then I have just divided the edge or selvage of the *frænum*, leaving the farther division to the action of the tongue; and by this method all danger has been avoided. Yet I believe it may be justifiable to say, that the *frænum* never requires cutting.

In so unhappy a case as that of a dangerous hæmorrhage occasioned by cutting the *frænum* of the tongue negligently, ignorantly, or rashly; it would perhaps be impossible to tye the vessels from which the blood was poured, and any application which could be safely employed might fail; yet one would hardly feel satisfied without trying, when all other means had failed, the actual cautery.

I am, &c.

*July* 9, 1805. THOMAS DENMAN.

